# Dehazing in hyperspectral images: the GRANHHADA database

**DOI:** 10.1038/s41598-023-46808-3

**Published:** 2023-11-13

**Authors:** Sol Fernández Carvelo, Miguel Ángel Martínez Domingo, Eva M. Valero, Javier Hernández Andrés

**Affiliations:** 1 Andalusian Institute for Earth System Research (IISTA), University of Granada, Granada, Spain; 2 Department of Applied Physics, University of Granada, Granada, Spain; 3 Color Imaging Lab, Department of Optics, University of Granada, Granada, Spain

**Keywords:** Optics and photonics, Physics

## Abstract

In this study, we present an analysis of dehazing techniques for hyperspectral images in outdoor scenes. The aim of our research is to compare different dehazing approaches for hyperspectral images and introduce a new hyperspectral image database called GRANHHADA (GRANada Hyperspectral HAzy Database) containing 35 scenes with various haze conditions. We conducted three experiments to assess dehazing strategies, using the Multi-Scale Convolutional Neural Network (MS-CNN) algorithm. In the first experiment, we searched for optimal triplets of spectral bands to use as input for dehazing algorithms. The results revealed that certain bands in the near-infrared range showed promise for dehazing. The second experiment involved sRGB dehazing, where we generated sRGB images from hyperspectral data and applied dehazing techniques. While this approach showed improvements in some cases, it did not consistently outperform the spectral band-based approach. In the third experiment, we proposed a novel method that involved dehazing each spectral band individually and then generating an sRGB image. This approach yielded promising results, particularly for images with a high level of atmospheric dust particles. We evaluated the quality of dehazed images using a combination of image quality metrics including reference and non-reference quality scores. Using a reduced set of bands instead of the full spectral image capture can contribute to lower processing time and yields better quality results than sRGB dehazing. If the full spectral data are available, then band-per-band dehazing is a better option than sRGB dehazing. Our findings provide insights into the effectiveness of different dehazing strategies for hyperspectral images, with implications for various applications in remote sensing and image processing.

## Introduction

In outdoor scenes, atmospheric phenomena such as fog or haze can cause problems with object identification or related tasks.

The main differences between fog and haze are the particle size and their concentration^1,2^. In this study, for simplicity’s sake we will refer to both fog and haze as haze from now on. When light passes through the atmosphere, scattering and absorption phenomena produce a reduction in intensity, which increases with the distance between the object and the observer (camera)^3,4^. Thus, images captured under haze are affected by a reduction in contrast and visibility, as well as by color alteration^5^. Depending on the purpose for which the images are used, overcoming this problem can be quite critical. That is why dehazing techniques started to be developed, being a far-reaching field of research in a continuous process of improvement. Their application domains are flight and navigation^6^, driving assistance^7,8^, face recognition^9^, mobile devices^10^, remote sensing^11,12^ or medicine^13^ among others. These techniques aim to eliminate or reduce the degradation of outdoor images, and consequently improve their quality. The dehazing algorithms can be classified according to whether they use a single image or multiple images^14^. Other classifications distinguish between methods that are based on physical models and methods that are not, the latter including those that use deep learning (combining different strategies in network design and training) or post-processing^15^. Although all these strategies have been thoroughly researched and developed, probably the most widely used single-image strategies are currently those based on deep learning^16^.

Driven by the need for data to test dehazing approaches, the number of image databases that contain simulated or real hazy images has also grown sustainedly. Building a database for dehazing applications may have inherent difficulties if it aims for providing ground-truth as well (i.e., images with no haze at all). The ideal situation would be that the completely dehazed image would be identical to the reference image captured in clear conditions. But this will be very rarely the case given the variability in atmospheric conditions. Therefore, the use of both computationally simulated haze and machine-generated haze databases has become a widespread procedure in the field of dehazing techniques. If we focus on the procedure to obtain the hazy image, we can divide the datasets into three main blocks: 1) databases where haze has been computationally simulated such as FRIDA, FRIDA2, FROSI, Foggy Cityscapes and Foggy Cityscapes Dual-reference cross-Bilateral Filter (Cityscapes), D-HAZY, HazeRD, RESIDE (ITS, SOTS and OTS)^17^, LIVE Image Defogging Database Release^18^, MRFID Image Defogging^19^ or Waterloo IVC Dehazed Image Database^20^; 2) databases where haze has been artificially generated by machines such as CHIC, SHIA, I-HAZE, STF, and NH-HAZE^17^ and 3) real image databases such as Foggy driving and Foggy Zurich (Cityscapes), O-HAZE, RESIDE (HSTS and RTTS), RADI-ATE, FOVI, DAWN and HUDRS^17^, BeDDE and exBeDDE^21,22^, WILD, Dense-Haze and AMOS^14^, NWPU-RESIC45, NWPUVHR-10, DOTA and RSOD^23^, FINEDUST^24^ or RGB/NIR Data Set^25^. Among all these, only the SHIA database^26^ contains hyperspectral image data from two very similar indoor scenes where the haze was artificially generated with a machine in an indoor environment and under fully controlled conditions, and RGB/NIR Data Set^25^ contains images captured with two devices, a RGB camera and an infrared capture device. Hyperspectral images have proven to outperform RGB images in many instances of image processing tasks including dehazing^26,27^, making it worth the additional effort required due to longer capture times and expensive capture devices. Despite this fact, hyperspectral image data are not commonly used for dehazing.

In a previous study^27^, the search for an optimal triplet of spectral bands that could be used as input for the dehazing algorithms was carried out using the SHIA database^26^, which has only two indoor scenes with artificially generated fog. The aim was to show how from the complete spectral information, one could choose to simplify the capture process with comparable results in terms of efficiency in the dehazing process. In this study, we use a new hyperspectral image database with 35 scenes including no haze, natural haze and simulated haze conditions. The focus of our research is to compare different approaches for dehazing hyperspectral images with one single algorithm. We introduce a new method for dehazing hyperspectral images which is performing a dehazing for all the bands in the spectral image and then generate a rendered sRGB dehazed image. This method will be compared with other two approaches previously used in^27^, which are the optimal triplet of bands and simple sRGB dehazing using sRGB renditions of the hyperspectral images. To deal with the case of real haze images for which a reference haze-free image is not available, we introduce an adaptation of the combined quality metric introduced in^27^ that uses non-reference image quality metrics as its main components. The new hyperspectral image database is called GRANHHADA (acronym for GRANada Hyperspectral HAzy DAtabase) and contains a total of 35 scenes, available for public access through.

To show the potential of this new database, we aim to find the best strategy for obtaining dehazed images in the GRANHHADA database, testing three different approaches: the first one is to find optimal triplets of bands for dehazing; the second one is generating sRGB rendered images and dehazing them; and the third one is performing a dehazing for all the bands in the spectral image and then generate a rendered sRGB dehazed image. The dehazed images produced in the second and third approaches can be directly compared, while the optimal triplets will not in general be visually comparable with standard sRGB images. To perform the dehazing, we have chosen the MSCNN algorithm, based on deep learning and widely used^28,29^. The main contributions of this work are: the proposal of a new database of hyperspectral images captured outdoors with simulated and real haze; the discussion about the best procedure for dehazing hyperspectral images; and the evaluation of results using a new combined metric proposed constructed from three of the best-known non-reference metrics. This paper is organized as follows: Section "Method" describes the new database and the haze simulation model used, the quality assessment metrics used and the search methodology for the best triplet; Section "Results" shows the analysis of the results and the comparison between the different strategies followed; and finally, Sect. 4 summarizes the most relevant conclusions and possibilities for future work.

## Method

### Granada hyperspectral hazy database (GRANHHADA)

#### Outdoor scenes capture

The GRANHHADA database includes 35 outdoor scenes which were captured at different locations near the city of Granada (Spain) and under different atmospheric conditions. Granada city has a latitude of 37°10′41″N, a longitude of 3°36′03″W, and an altitude of 684 m. Two capture devices which cover either visible and near-infrared (VIS/NIR, from 397 to 1004 nm) or only near-infrared (SWIR, from 900 to 1700 nm) were used. Fifteen scenes were captured on a very clear winter day without any fog or haze. Ten of them (five in the VIS/NIR range and five in the SWIR range) were captured from the observation tower of the Parque de las Ciencias Museum (50 m high). The remaining five scenes in clear conditions were captured from an elevated location above the village of Las Gabias. Twenty additional scenes were captured in presence of haze of natural origin and in the VIS/NIR range: ten of them from an elevated location above the city of Granada; seven from the rooftop of a building in Granada and thirteen from the elevated location above the village of Las Gabias. These scenes have been used to obtain the simulated hazy images in the database, with the procedure explained in section "Haze simulation".

The capture device used for the VIS/NIR range was a SPECIM-IQ hyperspectral camera^30^ based on a CMOS sensor with 512 × 512 pixels spatial resolution, with 204 bands. For each scene, a raw capture of the scene and a raw capture of a reference white sample as well as a dark image were captured. With this information, the spectral reflectance image was then calculated by correcting each pixel of the raw image by applying a flat field correction, such that:

1 $${Cube}_{ref}=\frac{\left({Cube}_{raw}-{Cube}_{dark}\right)}{\left({Cube}_{white}-{Cube}_{dark}\right)}\times {white}_{ref}$$

where Cube_raw_ refers to the raw capture of the scene, Cube_white_ to the raw capture of the white sample, Cube_dark_ to the dark image, *white*_*ref*_ to the spectral reflectance of the white reference tile used to capture *Cube*_*white*_, and Cube_ref_ is the corrected reflectance cube.

In the SWIR range (from 888 to 1732 nm), the capture device used was a PIKA NIR-640 hyperspectral camera^31^. This camera is based on a linear sensor (InGaAs) with 640 pixels of spatial resolution and 336 bands, which we reduced to 168 using spectral binning (averaging between adjacent bands).

This camera is radiometrically calibrated to convert raw sensor responses to radiance, so the radiance cube is obtained directly.

For the experiments in this study, we have used the thirty images in the VIS/NIR range. The images in the SWIR range have not been used for the experiments described in the following sections for brevity’s sake, but they are included in the GRANHHADA database and can be of potential interest for the scientific community. We have divided the VIS/NIR used in the experiments into two subsets: image subset 1 (10 scenes) is formed by images with simulated haze, and image subset 2 (20 scenes) by images with real haze. The two subsets will be analyzed separately.

#### Haze simulation

Due to the low ambient humidity and the prevalence of light winds, the annual rate of foggy days in Granada is remarkably low. For this reason, and for the intrinsic interest of being able to introduce different conditions in the database, we have used image subset 1 to simulate haze based on the dichromatic atmospheric spreading model^32^. This is a commonly used method for haze simulation^33–39^. The dichromatic model combines two terms (direct transmission and airlight). The spectral radiance of the image at each scene point in the simulated hazy scene, *I(x,λ)*, is then obtained as:

2 $$I\left(x,\lambda \right)=\left({L}_{0}(x,\lambda )\times {e}^{-\beta \left(\lambda \right)d(x)}\right)+\left({L}_{\infty }(x,\lambda )\times \left(1-{e}^{-\beta \left(\lambda \right)d(x)}\right)\right)$$

where *x* = *(x*_*1*_*,x*_*2*_*)* is the vector containing the 2D pixel coordinates in the image, λ is the wavelength, *L*_*0*_*(x,λ)* is the spectral radiance in the original scene, *L*_*∞*_*(x, λ)* is the spectral radiance from the horizon, *β(λ)* is the atmospheric extinction coefficient, and *d(x)* the distance between the object in spatial location x and the capturing device. The values for *β(λ)* in the visible range have been provided by The Andalusian Global Observatory of the Atmosphere (AGORA). These values are automatically recorded daily for three wavelengths (450, 550 and 700 nm) in the Interuniversity Institute of the Earth System in Andalusia, IISTA-CEAMA (Granada), using an integral Nephelometer (TSI, model 3563).

**Figure 1:**
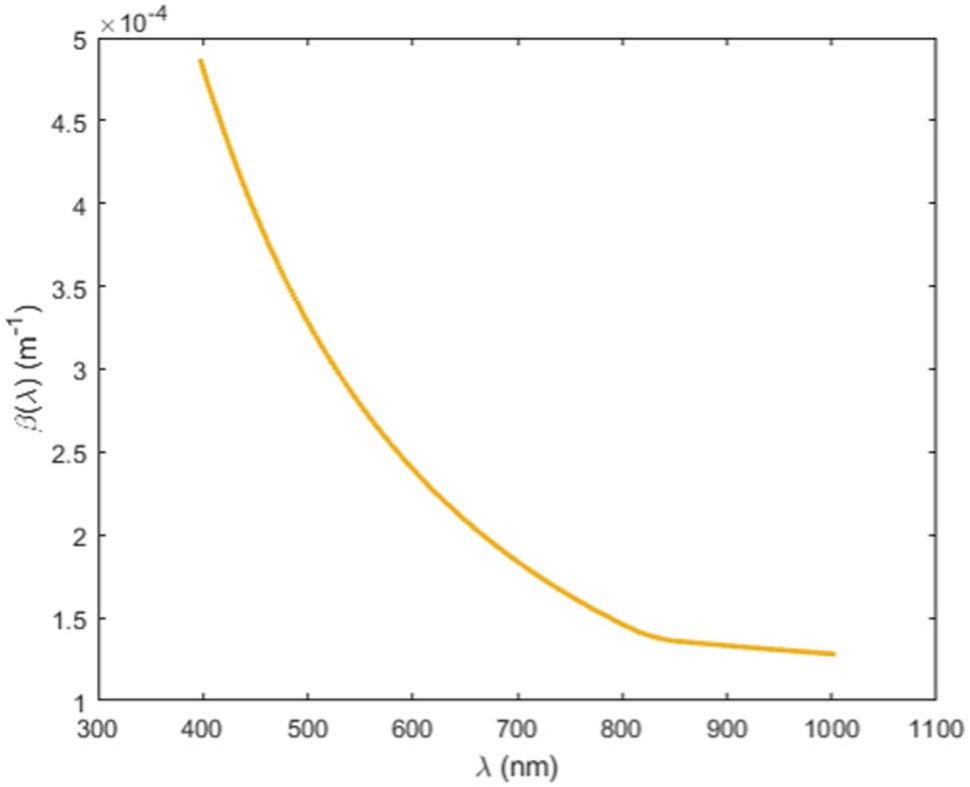
Extinction coefficient values interpolated between 400 and 1000 nm, using the recorded data on November 20, 2021, 08:44 AM for wavelengths 450, 550 and 700 nm by AGORA, and the extrapolation to the NIR range from Pachenko et al. data^40^.

To make the simulation as realistic as possible, the representative values for β(λ) correspond to a day and time where the presence of haze in the city was clearly noticeable (November 21, 2021, 08:44 AM). For wavelengths above 700 nm, the data collected in the city of Tomsk and the NIR range by Pachenko et al.^40^ were used to obtain a trend to extrapolate our extinction coefficient curve to the 700–1000 nm range. Thus, the extinction coefficient from 400 to 700 nm corresponds to the data recorded in Granada and from 700 to 1000 nm it follows the trend of the data recorded in Tomsk under smog conditions in autumn (see Fig. 1). To ensure a seamless transition between both sets, the NIR range data were adjusted by vertically displacing the linear fit until the value for 700 nm was equal to the visible value measured in Granada.

**Figure 2:**
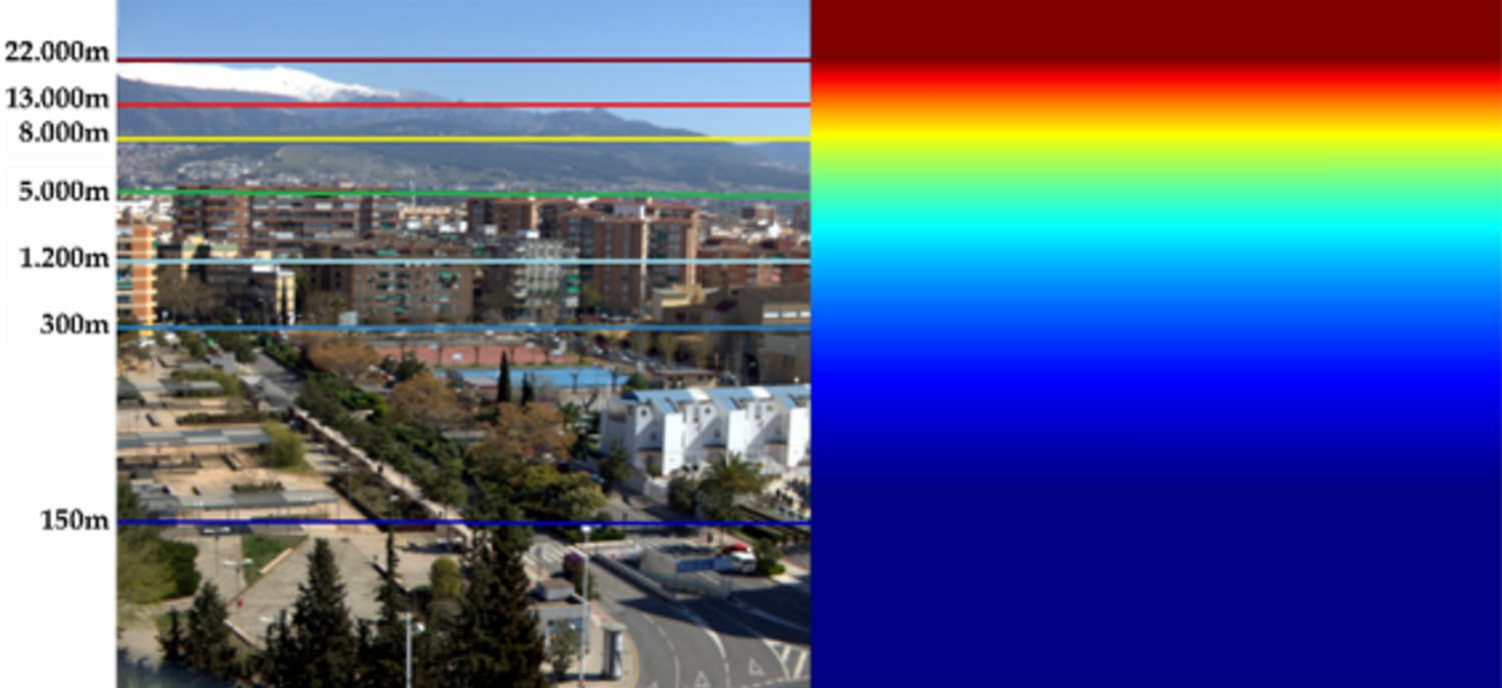
Example of one of the scenes with simulated haze (false RGB contained using bands which correspond to 645, 563 and 440 nm) and distance map generated by the linear adjustment for that scene.

To obtain the distance distribution (d(x) in Eq. 2), we have used a simple vertical distance gradient model formed by using adjacent linear fits, which assumes that all points in the scene that lie on the same horizontal line are at the same distance from the camera. This assumption does not hold in all cases, but it does generally work fine as an approximation. We have located singular buildings and Google Earth provided the distance in a straight line between the buildings and our camera location. With these data, we were able to build the initial distance data distribution for each scene, and then interpolate the values in between distance markings. To obtain the distance gradient data, different singular buildings and points of interest in the city have been identified and located using Google Earth software. All the pixels along the line of these singular elements have been assigned the same distance value (see Fig. 2). A piecewise linear fit has been used to obtain the missing d(x) values for the lines between each two singular elements in the scene. For instance, the first piecewise linear fit in Fig. 2 is the line that passes through points (0,150) and (N, 300), where N = Y_2_-Y_1_, being Y_2_ the vertical pixel position of the 300 m horizontal mark in Fig. 2 and Y_1_ the vertical pixel position of the 150 m horizontal mark. This line is displaced in the horizontal axis to start at Y_1_ value, and the pixels with lower Y pixel positions are assigned a fixed d(x) value of 150 m. The following restrictions have been imposed: 1) the objects above the farthest singular element are at equal distance from the camera, and 2) the points below the nearest singular element are at equal distance from the camera. The horizon radiance value has been set to 1 (pure white), which is a common practice in haze simulation in previously published work^33^.

The main limitations of the simulated haze model used are the vertical gradient model with the two restrictions in the extremes of the distance range, and the spatial homogeneity of the simulated haze. We are aware of these limitations, but we are still interested in keeping a simple simulation model to be able to discuss the differences with the real hazy scenes in the database. Any of the scenes captured in clear conditions can be used to apply more sophisticated haze simulation models by anyone interested in testing them, but this was not among the aims of our study.

### Image quality metrics

Different image quality assessment (IQA) strategies can be used to evaluate the visual quality of an image, either objectively or subjectively^15^. In the context of dehazing, higher quality means less haze present in the image and more objects that can be distinguished. Depending on the availability and/or type of the reference image, objective assessment metrics can be divided into three groups: full reference metrics^41–47^, reduced reference metrics^48–51^ and non-reference metrics^17,52–57^. When the reference image (haze-free image) is available, the metrics usually employed to evaluate the performance of dehazing algorithms are the full reference ones. In this work, we have used *CM-DIE*^26^, defined as shown in Eq. (3),

3 $$CM-DIE={W}_{1}\cdot \left(1-MSSSIM\right)+{W}_{2}\cdot \left|1-VIF\right|{+W}_{3}\cdot \left(MSiCID\right)$$

where *W*_*1*_, *W*_*2*_ and *W*_*3*_ are relative weights with values 0.5932, 0.3031 and 0.1037 respectively, for the metrics *MSSSIM*^42^, *VIF*^47^ and *MSiCID*^41^. The relative weights have been calculated using the average values of MSSSIM, VIF and MSiCID for image subset 1. Given that MSSSIM_m_, VIF_m_ and MSiCID_m_ are the average values of the metrics SSIM, VIF and MSiCID for the images contained in image subset 1, to calculate the weights W_1_ , W_2_ ·and W_3_ ·we impose the following conditions: W_1_ · (1 − MSSSIM_m_) = W_2_ · |1-VIF_m_|= W_3_ · (MSiCID_m_), and W_1_ + W_2_ + W_3_ = 1. Since the weights W_i_ have been calculated specifically for image subset 1, if the metric were to be used for different images or different distortions (for instance, image compression or noise), then the weights should be re-computed using the same procedure described above. The metric could then be used (with different weights) for other applications, with the same design principle. The closer the value of CM-DIE is to 0, the more similar the two images compared are. An image exactly equal to the reference image results in CM-DIE values of zero. However, the upper limit is not defined because it depends on the maximum values of the three components for image subset 1. This upper limit then cannot be defined in an absolute way, but it is dependent on the particular set of images used to compute the weights.

The full reference metrics cannot be used when haze free images are not available. Therefore, for the 20 images of image subset 2, we have used three non-reference metrics to build a combined new quality metric on the same principle as CM-DIE: Blind/reference-less Image Spatial Quality Evaluator (BRISQUE)^53^, Natural Image Quality Evaluator (NIQE)^54^ and Perception-based Image Quality Evaluator (PIQE)^55^. In all three cases, the lower the value of the metric, the better the quality of the evaluated image. The new metric is called CNM-DIE (Combined Non-reference Metric for Dehazed Image Evaluation), and it is defined as:

4 $$\text {CNM-DIE} = {W}_{4}\cdot \left(BRISQUE\right) +{W}_{5}\cdot \left(NIQE\right)+ {W}_{6}\cdot \left(PIQE\right)$$

where W_4_, W_5_ and W_6_ are relative weights with values 0.1008, 0.8226 and 0.0766 respectively. These weights are computed using the average values of the three-component metrics for the scenes in image subset 2, and the condition that W_4_ · (BRISQUE_m_) = W_5_ · (NIQE_m_) = W_6_ · (PiQE_m_). The closer to 0 the value of CNM-DIE is, the higher the quality of the evaluated image. The range of values for the CNM-DIE metric is higher than for CM-DIE.

## Results

We have performed three dehazing experiments using 30 images of the GRANHHADA database. As explained in section "Outdoor scenes capture", the images are divided into two subsets: image subset 1 contains 10 images with simulated haze, and image subset 2 contains the 20 images with real haze. The quality of the dehazing results for all the experiments will be evaluated using CM-DIE and CNM-DIE for image subset 1, and CNM-DIE for image subset 2. We will describe each experiment and the results obtained for the two image subsets in the next subsections.

### Experiment 1: optimal triplet search

#### Experiment 1 workflow

**Figure 3:**
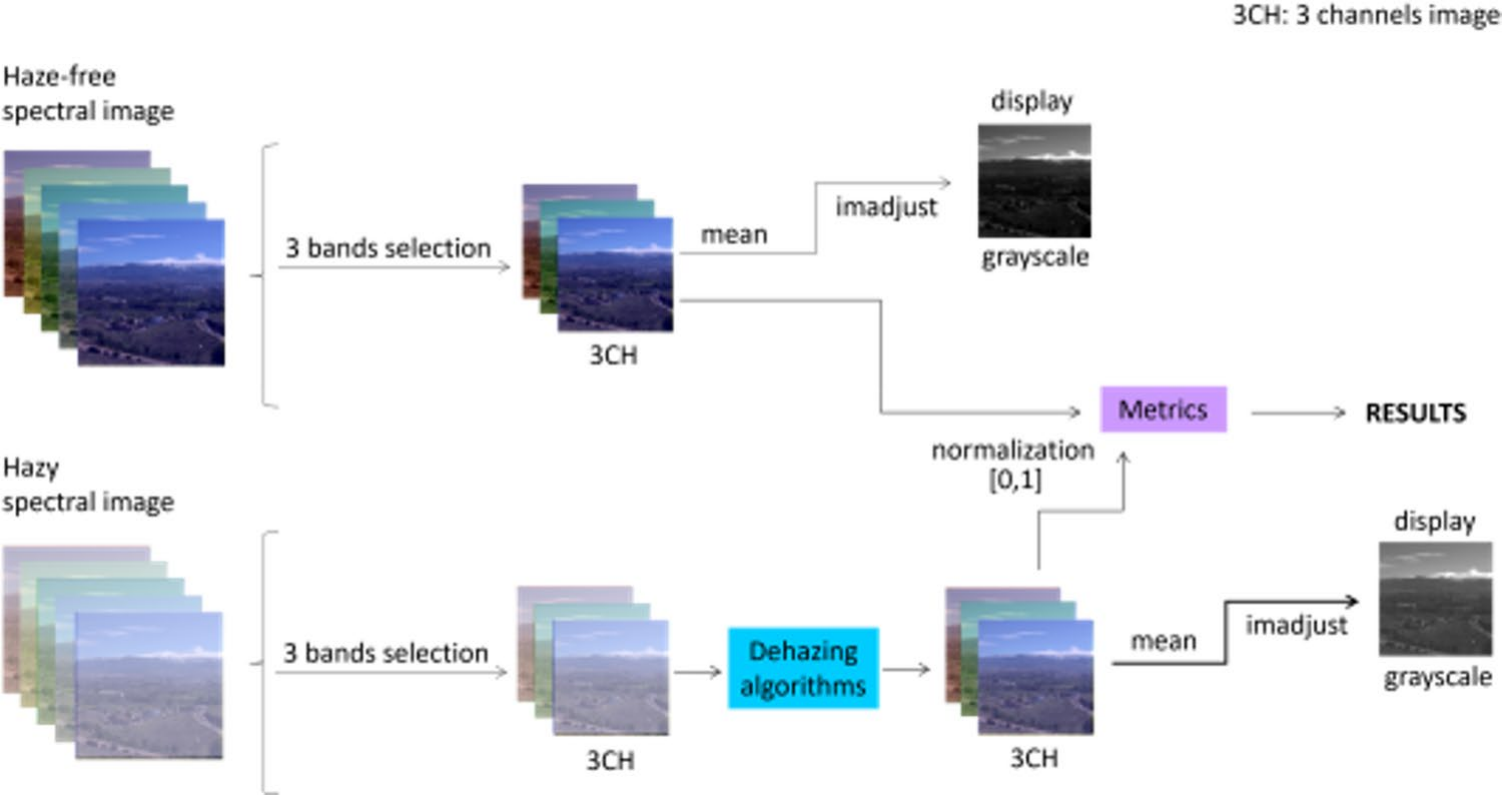
Workflow for experiment 1. Upper row: haze-free image. Lower row: hazy image. 3CH means three-channel image, formed by the candidate band triplet. The imadjust function from Matlab is used to enhance the contrast of grayscale/sRGB images in order to better visualize some details on them.

In this experiment, we will select triplets of bands from the full spectral images of the GRANHHADA database and evaluate the results of dehazing with MS-CNN by calculating the CM-DIE and/or CNM-DIE values. The optimal triplet is found by brute force from a set of candidates. For each candidate triplet and each image, dehazing is performed and the quality metrics are evaluated. The best triplet for each image subset and metric is the one with the lowest quality metric value in average for the image subset used. The workflow of this experiment is shown in Fig. 3 for image subset 1, and it is very similar to the procedure used for optimal triplet search in^27^. We start with the full hyperspectral image, then a subset of three bands is selected with the restrictions explained below. The average grayscale image is generated and enhanced (just for visualization purposes) using the ‘imadjust’ function in Matlab. This function adjusts the intensity values in the grayscale image, thereby increasing its contrast. By default, it extends the intensity range by saturating the bottom 1% and the top 1% of pixel values in the image. This prevents the image from appearing overly dark or light. The three channel images are normalized to have their values ranging between 0 and 1, previous to the image quality metrics computation. The workflow is identical for the haze-free and hazy images, save for the dehazing algorithm that is used on the second. The grayscale images are generated just for visualization of results, but the dehazing quality is computed from the dehazed triplets.

To obtain the set of candidate triplets, we start from a spectrally cropped version of the hyperspectral image, containing 198 channels. We have suppressed the first 6 channels due to the low responsivity of the Specim IQ camera CMOS sensor. Then, we build the set of candidate triplets using the following restrictions: a) initial set of bands with wavelengths differing by at least 6 nm; b) bands ordered from longest to shortest wavelength following the order of the channels of a conventional RGB image; c) a minimum distance of 100 nm between adjacent channels. This way, the possible combinations have been reduced from 7,880,599 (with no restrictions) to 13,671, allowing a shorter run time for the evaluation of the performance of all combinations. As explained before, the triplet among the 13,671 that obtains the lowest quality metric values is considered the optimal triplet.

#### Experiment 1 results

Image subset 1 is affected by simulated haze as explained in section "Haze simulation", while image subset 2 contains real haze with some atmospheric dust in suspension. In Table 1, the bands and metric values corresponding to the optimal triplets found for each metric and image subset are shown, along with the standard deviation, the mean, the median and the range covered by the average metrics values across the images for each subset.

**Table 1:** Optimal bands and optimal metric values found for the optimal triplets for each image subset and metric. The mean and standard deviation of the metric values for the full set of triplets is indicated in parenthesis in the third column, the median in the fourth column and the range of values in the fifth column.

	Optimal bands (nm)	Metric optimal value (STD)	Metric mean value	Metric median value	Metric range
Subset 1 CM-DIE	988, 887, 787	0.342 (0.097)	0.429	0.432	0.342–0.495
Subset 1 CNM-DIE	951,850, 643	10.800 (0.999)	11.687	11.671	10.800–12.761
Subset 2 CNM-DIE	924,760, 414	8.274 (1.185)	9.484	9.566	8.274–10.168

The optimal triplet found depends to some extent on the metric used, but the trends found are similar for all three components of the compound metrics. For image subset 1, the three wavelengths with the highest frequency of occurrence in the optimal triplets are above 780 nm for CM-DIE, and somewhat lower for CNM-DIE, but still covering from red to near infrared. It is not surprising to find that MS-CNN performs better in the infrared range because the radiation penetrates more into the haze and, therefore, the input images tend to be less hazy. For image subset 2, only one of the three most frequently selected wavelengths is in the NIR region. The results are then different from the ones obtained for image subset 1, but the type of haze is also different, and this influences the results of the dehazing as well. The haze affecting image subset 2 is formed by dust red particles, which scatter the red wavelengths significantly. The mean and median values across triplets are similar in all cases, but only for subset 1 and CNM-DIE the median is below the mean. The relative variation shown by the range of values (sixth column of Table 1) is higher for CM-DIE than for CNM-DIE, showing a trend for higher data dispersion in this metric.

**Figure 4:**
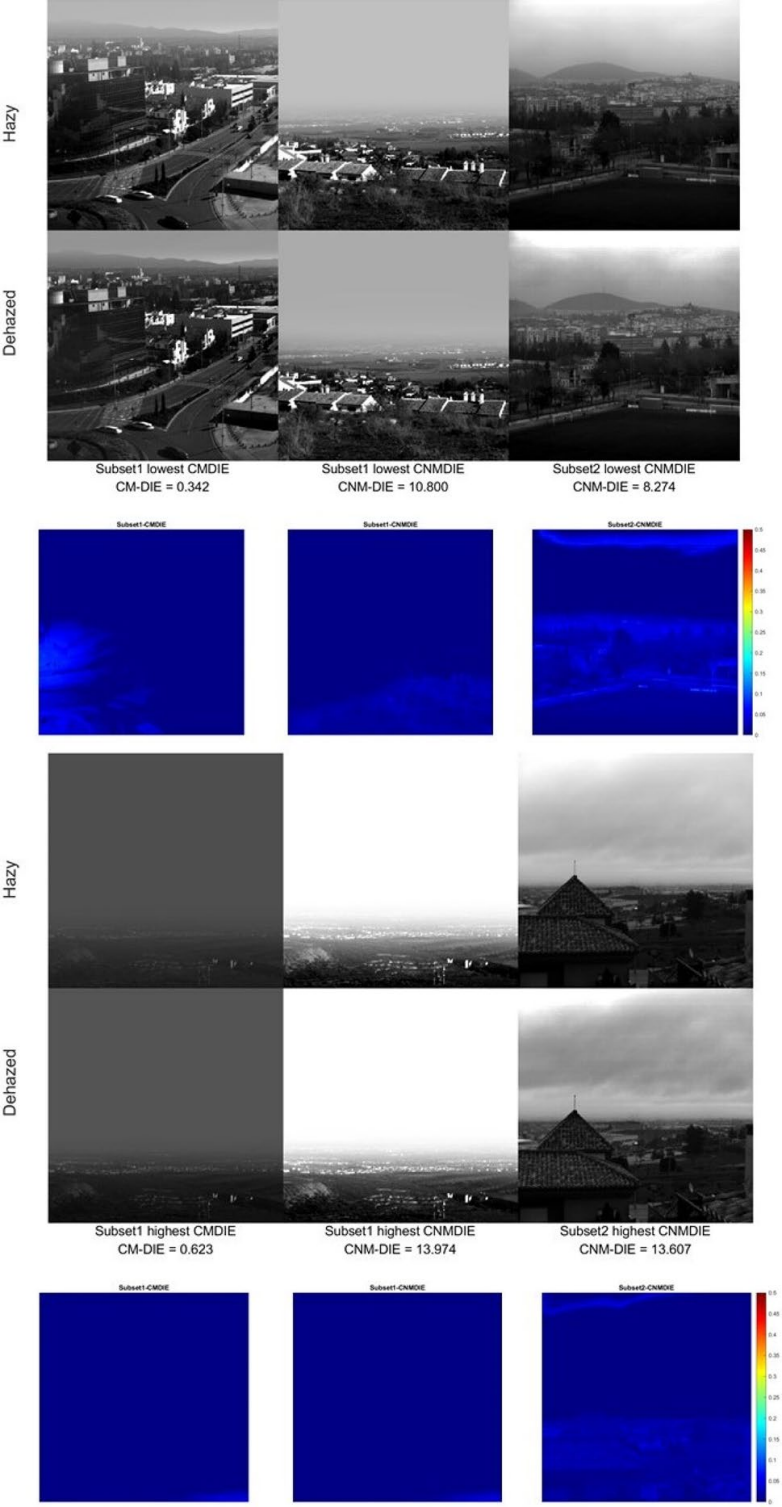
Best case scenario (above) for the two subsets and the two metrics tested: hazy and dehazed images for the optimal triplet which resulted in the lowest metric value among the images in each subset. Worst case scenario (below): highest metric values found for each subset and metric (always with the optimal triplet). The third row of images shows the difference images displayed in false color.

As it has been proved in previous studies, there is not always a satisfactory correlation between the quality metrics and visual evaluation^58^. Therefore, we have also generated grayscale images of the optimal triplets for each algorithm and each scene. Since the optimal triplets are different for each subset and metric, using false RGB images with these triplets would make a direct visual comparison very inaccurate. In Fig. 4, we show some of these images corresponding to the best- and worst-case scenario among the images tested, along with a difference image between hazy and dehazed displayed with a heat map false color scale.

In Fig. 4 above left, we see a noticeable effect of the dehazing in the mountains profile and the buildings in the mid-distance central region of the image. The effect is less noticeable for the other two images (center and right), because the haze is less noticeable in the hazy image as well. The difference images in false color show also some effect of the dehazing in the middle and near distance portion of the images, which are less perceptible when comparing directly the hazy and dehazed scenes. In the worst cases (Fig. 4 lower row), there is little difference between hazy and dehazed images (save for the left and central image in the mid-distance portion of the scene), and the further distance regions in the images do not register any change. In the difference images, it is observed how the effect of the dehazing is more apparent for the scene on the right, belonging to image subset 2. These worst cases correspond to scenes in which the haze is high or there is cloud cover in more than half of the scene. Since haze strongly depends on distance and our scenes include a wide range of distances, the effects of dehazing tend to be more noticeable in the mid-portion section of the scenes, because for the near distances there is not enough amount of haze, while for the long distances in some cases there tends to be too much haze for the dehazing algorithm to work effectively. In general, we can say that there is a certain correlation between the metrics’ results and a visual assessment of the quality of the dehazed images.

### Experiment 2: sRGB dehazing

In this experiment, we will use the visible range of the hyperspectral scenes to build an sRGB image, and then perform the dehazing. This way, we will be able to compare our previous results using only spectral information, with the standard way in which the dehazing algorithms are designed to operate, i.e., using RGB images as input.

#### Experiment 2 workflow

**Figure 5:**
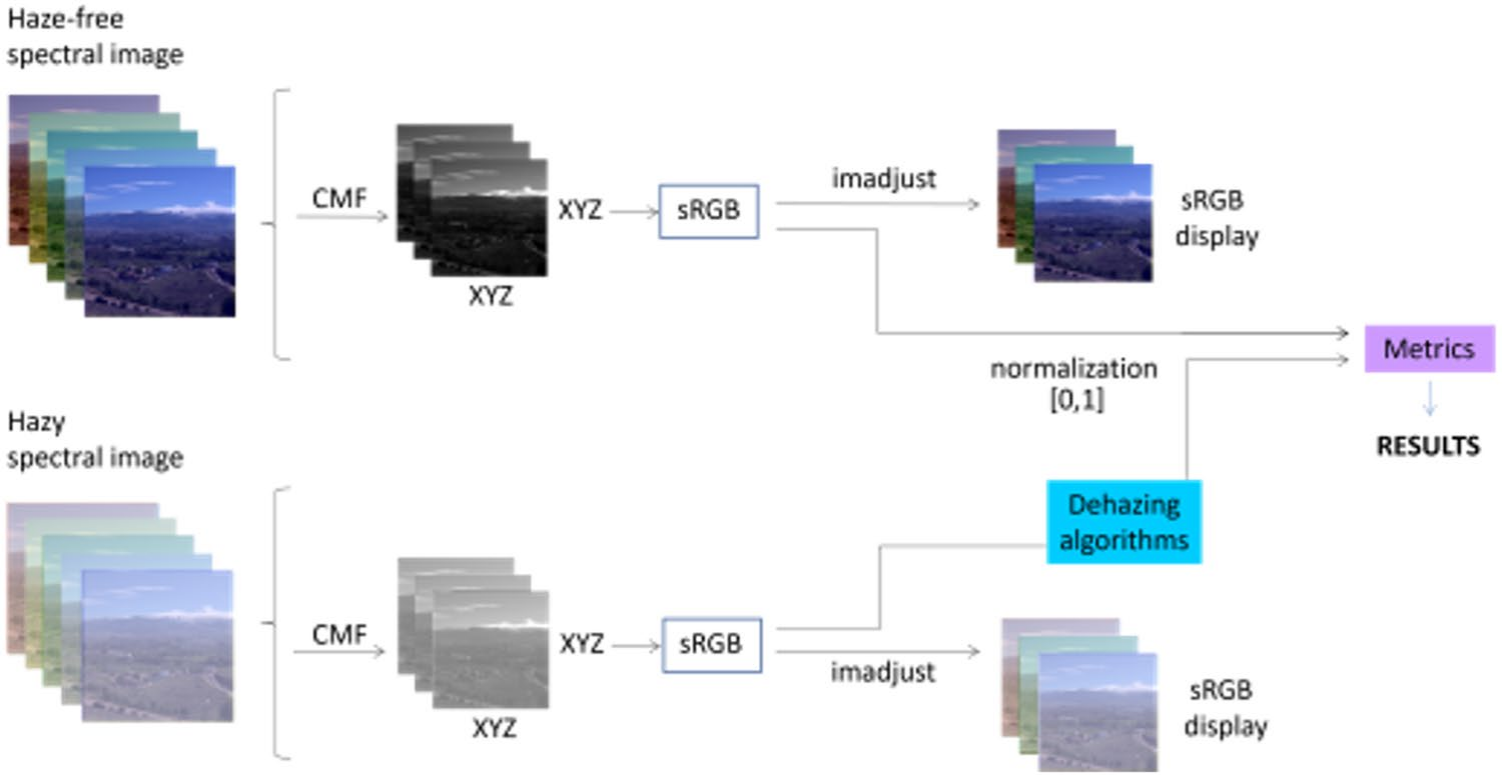
Workflow for experiment 2. Upper row: haze-free image. Lower row: hazy image.CMF: CIE Color-Matching Functions for the 2-deg standard observer. XYZ: CIE XYZ tristimulus values. The imadjust function from Matlab is used to enhance the contrast of grayscale/sRGB images in order to better visualize some details on them.

In Fig. 5 we show the schematic workflow for sRGB dehazing. We have used the spectral radiance at all wavelengths within the visible to generate the CIE 1931 XYZ tristimulus values, and from these, we have obtained an sRGB image using the standard transformation, that converts a spectral image, represented as spectral bands, into a color image. This conversion can be done in three different color spaces: XYZ, Lab, and sRGB. The resulting color image was also contrast-adjusted. In this case, each color channel (Red, Green, Blue) of the input color image is adjusted independently. The adjusted channels are then combined to create the output adjusted color image. This processing enhances the contrast of each channel individually, potentially leading to an overall improvement in the appearance of the color image. Thereafter, the images were normalized to [0,1] range for sRGB values. Then, we used the normalized hazy sRGB images as input for the dehazing algorithms and evaluated the quality of the result either with CM-DIE (image subset 1) or with CNM-DIE (image subset 2) by comparing with the reference normalized sRGB images. This experiment was also carried out with the images from the SHIA database in^27^. The sRGB images have been enhanced using the *imadjust* function in Matlab only for visualization purposes.

#### Experiment 2 results

Table 2 shows the mean, median and range of values as well as the standard deviation of the CM-DIE metric (image subset 1) and CNM-DIE metric (image subset 2).

**Table 2:** Mean values and standard deviation of the combined image quality metrics (second row); median values (third row) and range of values (fourth row) for both subsets and sRGB Dehazing.

	CM-DIE image subset 1	CNM-DIE image subset 1	CNM-DIE image subset 2
Mean values (std)	0.469 (0.093)	11.050 (1.336)	9.291 (0.814)
Median	0.459	11.035	9.192
Range	0.342–0.647	9.625–13.809	7.957–10.859

The metrics values tend to be higher for the sRGB images than in the previous experiment, showing that using optimal triplets could be a better strategy than standard sRGB dehazing. The lowest relative improvement in the metrics values when comparing the average values between the two experiments happens for CNM-DIE and image subset 1, with a relative improvement of 2.2%, while for image subset 1 and CM-DIE the relative improvement is 27%, and for image subset 2 the dehazing lowers CNM-DIE values by 10.94%. The median values tend to be slightly lower than the mean values, which means that the distribution of metric values is not significantly skewed. The range spanned by the metrics shows that there is a certain overlap between the quality distribution for experiment 1 and experiment 2: the lower part of the range of values for experiment 2 overlaps with the mean-to-higher range of values for experiment 1.

**Figure 6:**
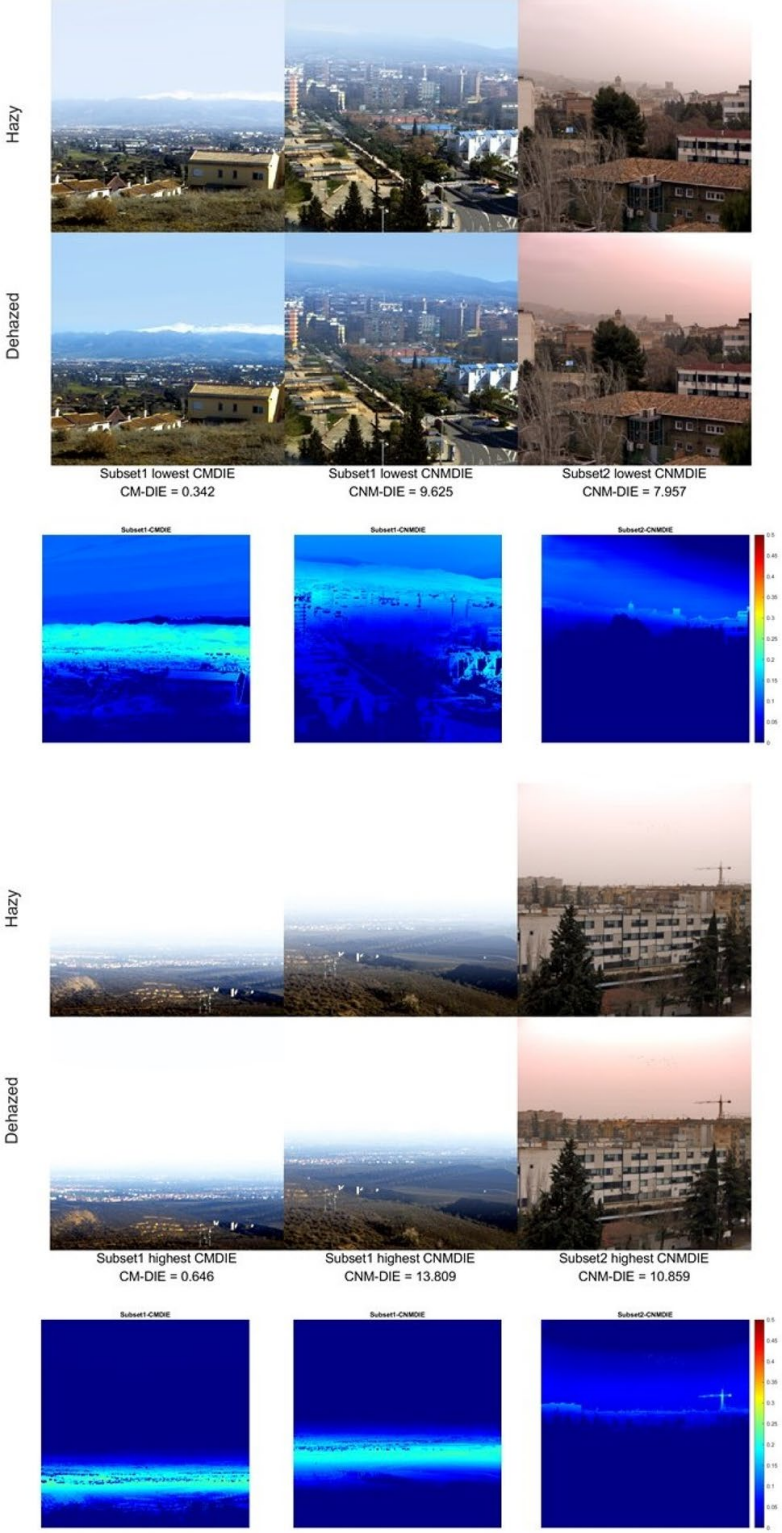
Best case scenario (above) for the two subsets and the two metrics tested in sRGB dehazing: hazy and dehazed images for the optimal triplet which resulted in the lowest metric value among the images in each subset. Worst case scenario (below): highest metric values found for each subset and metric. The third row of images shows the difference images displayed in false color.

In Fig. 6, the best and worst case scenarios along with the difference images in false color are shown for both subsets.

A noticeable improvement is found in the image contrast and visibility of middle and far range objects for the best cases (Fig. 6 upper row). In the worst cases (Fig. 6 lower row), again most or at least half of the image is covered by cloudy sky, with which the dehazing algorithm can do little to improve. Even with that limitation, there is a certain gain for the objects placed between near and mid-distance range, and for the best case scenario even in the mountain profiles as well, as shown by the difference images in false color.

### Experiment 3: mono-band full spectral dehazing and sRGB rendering

In this final experiment, we tested an alternative approach to dehazing that uses the full spectral image. We first performed the dehazing band by band with each monochrome image input for MS-CNN, then generated the sRGB rendering using the dehazed bands, and finally evaluated the result. Our hypothesis is that this approach will produce better results than the conventional sRGB dehazing shown in experiment 2.

#### Experiment 3 workflow

**Figure 7:**
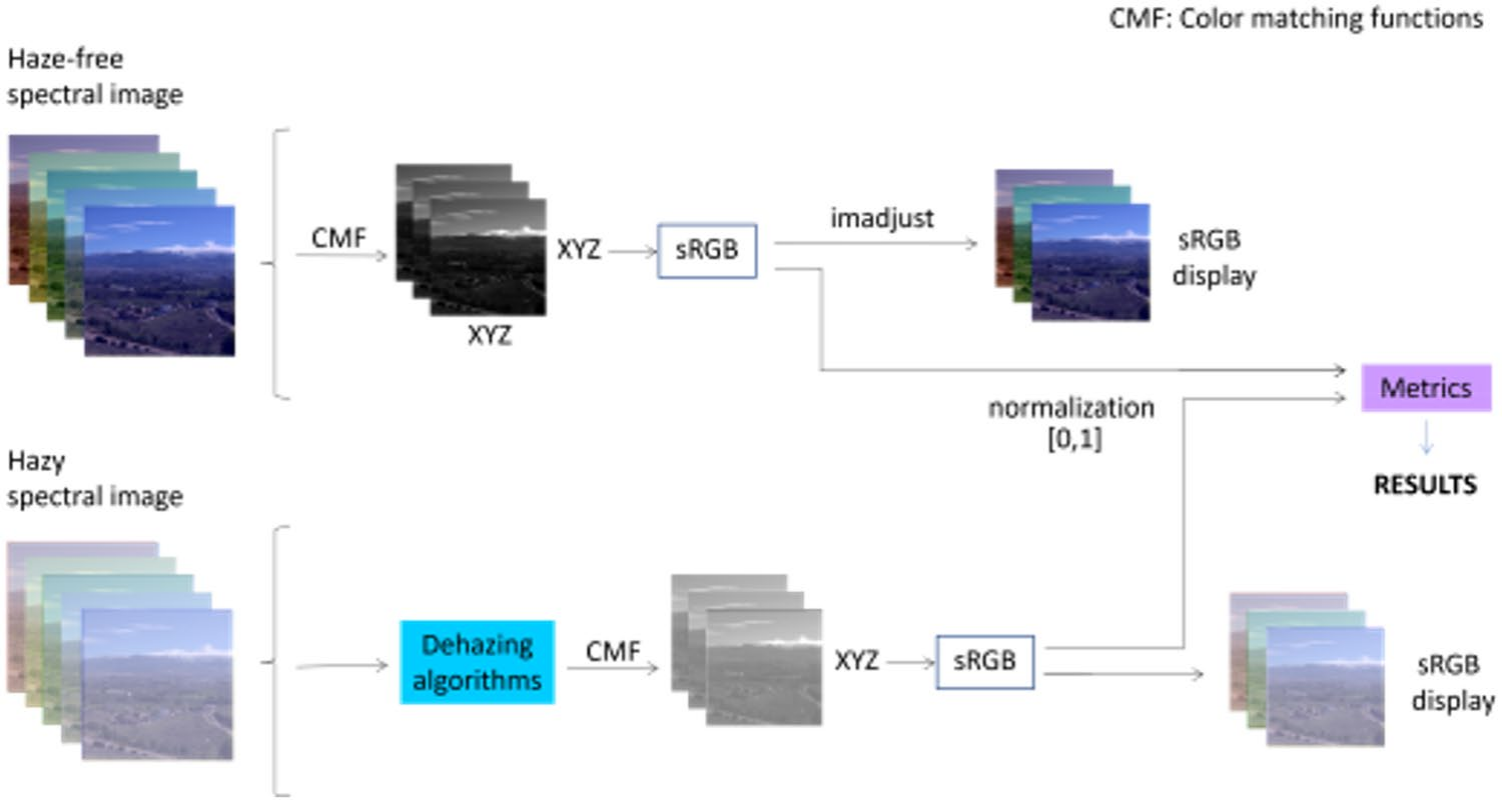
Workflow for experiment 3. Upper row: haze-free image. Lower row: hazy image.CMF: CIE Color-Matching Functions for the 2-deg standard observer. XYZ: CIE XYZ tristimulus values. The imadjust function from Matlab is used to enhance the contrast of grayscale/sRGB images in order to better visualize some details on them.

Figure 7 shows the final experiment workflow for image subset 1. The main difference with respect to Experiment 2 is the band-by-band dehazing step of the hazy spectral images before the XYZ and sRGB rendering steps (lower row of Fig. 7). This experiment was not performed in^27^, and shows a new way of performing dehazing for spectral images.

#### Experiment 3 results

Table 3 shows the average, median and range of metrics values and standard deviations obtained for each set and metric in this experiment.

**Table 3:** Mean values and standard deviation of the combined image quality metrics (second row); median values (third row) and range of values (fourth row) for both subsets and band-by-band dehazing previous to sRGB rendering.

	CM-DIE image subset 1	CNM-DIE image subset 1	CNM-DIE image subset 2
Mean values (std)	0.426 (0.104)	11.084 (2.029)	8.407 (0.835)
Median	0.405	10.786	8.471
Range	0.273–0.540	8.989–14.846	6.388–9.552

There is a small improvement from experiment 2 in subset 1 and CM-DIE metric, but with CNM-DIE for image subset 1 the metric values are practically the same, with higher standard deviation in general for this subset. The most relevant improvement happens for image subset 2 with a relative increase in quality (descent in CNM-DIE values) of 9.5%. It seems that for images with a high level of dust particles present in the atmosphere it might be a better approach to perform dehazing band-by-band using the spectral data, but there is relatively little gain in using this procedure for images with some clear areas in the foreground, like those in image subset 1. The trends described for experiment 2 regarding the median values are also present in this experiment, although the median for CNM-DIE and image subset 2 is slightly higher than the mean. There is also some overlapping in the ranges of the three metric values for experiment 2 and experiment 3.

**Figure 8:**
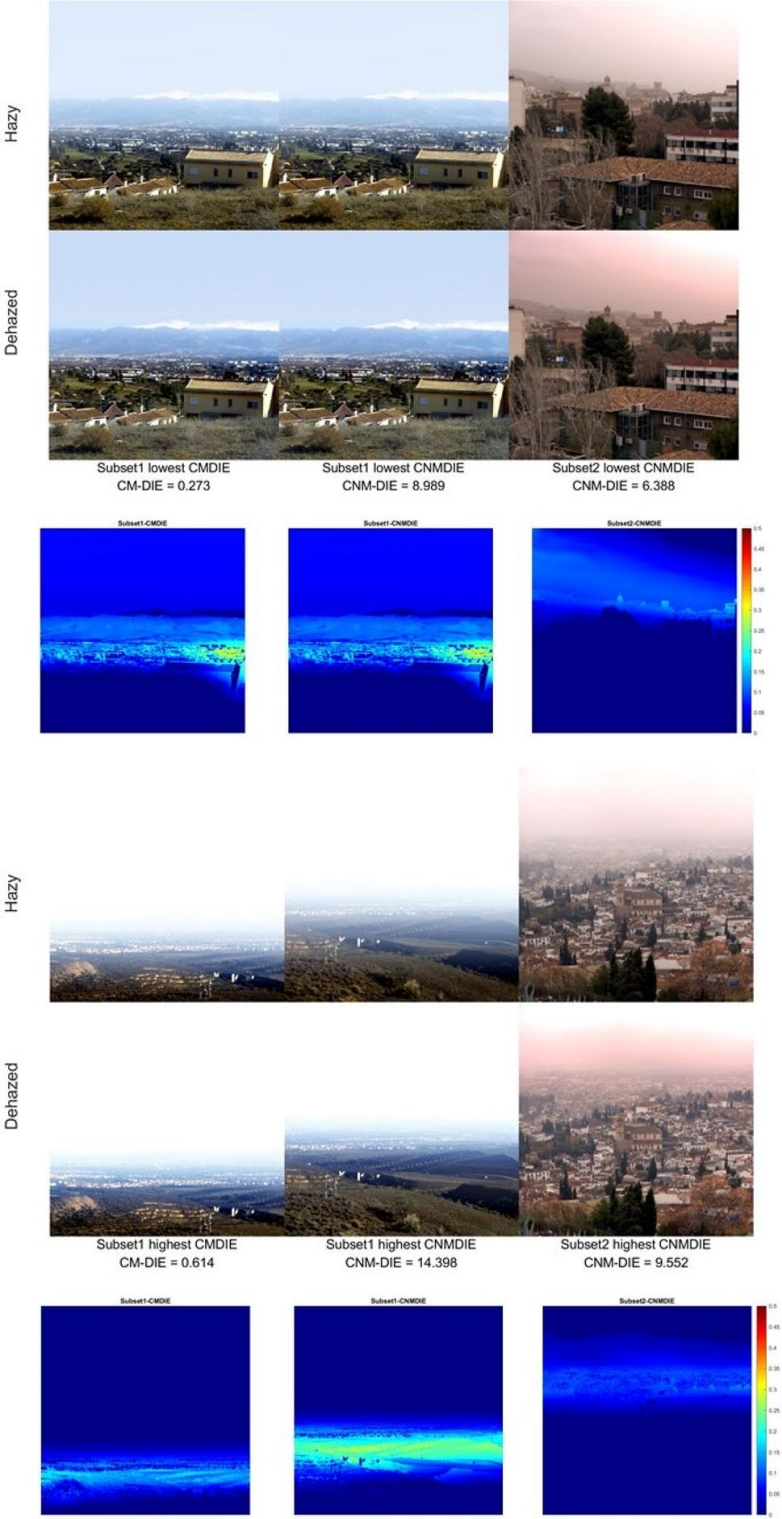
Best case scenario (above) for the two subsets and the two metrics tested in band-by-band dehazing and sRGB rendering: hazy and dehazed images for the optimal triplet which resulted in the lowest metric value among the images in each subset. Worst case scenario (below): highest metric values found for each subset and metric. *The third row of images shows the difference images displayed in false color.*

In Fig. 8, we show the analog of Fig. 6 for the dehazing strategy used in Experiment 3. Three among the six images are also present in the best and worst case scenarios for experiment 2, shown in Fig. 6. In the best cases, the improvement in the far distance range, for which the mountains of the Sierra Nevada range are visible (Fig. 8 left and center columns) is clearly noticeable, also when looking at the difference images. The worst case scenario corresponds to the same images shown in Fig. 6 for image subset 1, and for them the cloud cover and the amount of haze present in most of the image renders quite difficult the task of dehazing. For image subset 2, in the best case most of the image is in the near-to-mid range with less haze present, while the opposite happens in the worst case scenario, with an image containing much information in the mid-to-far distance range. Even in the worst case, we are able to see some noticeable changes in the difference images.

**Figure 9:**
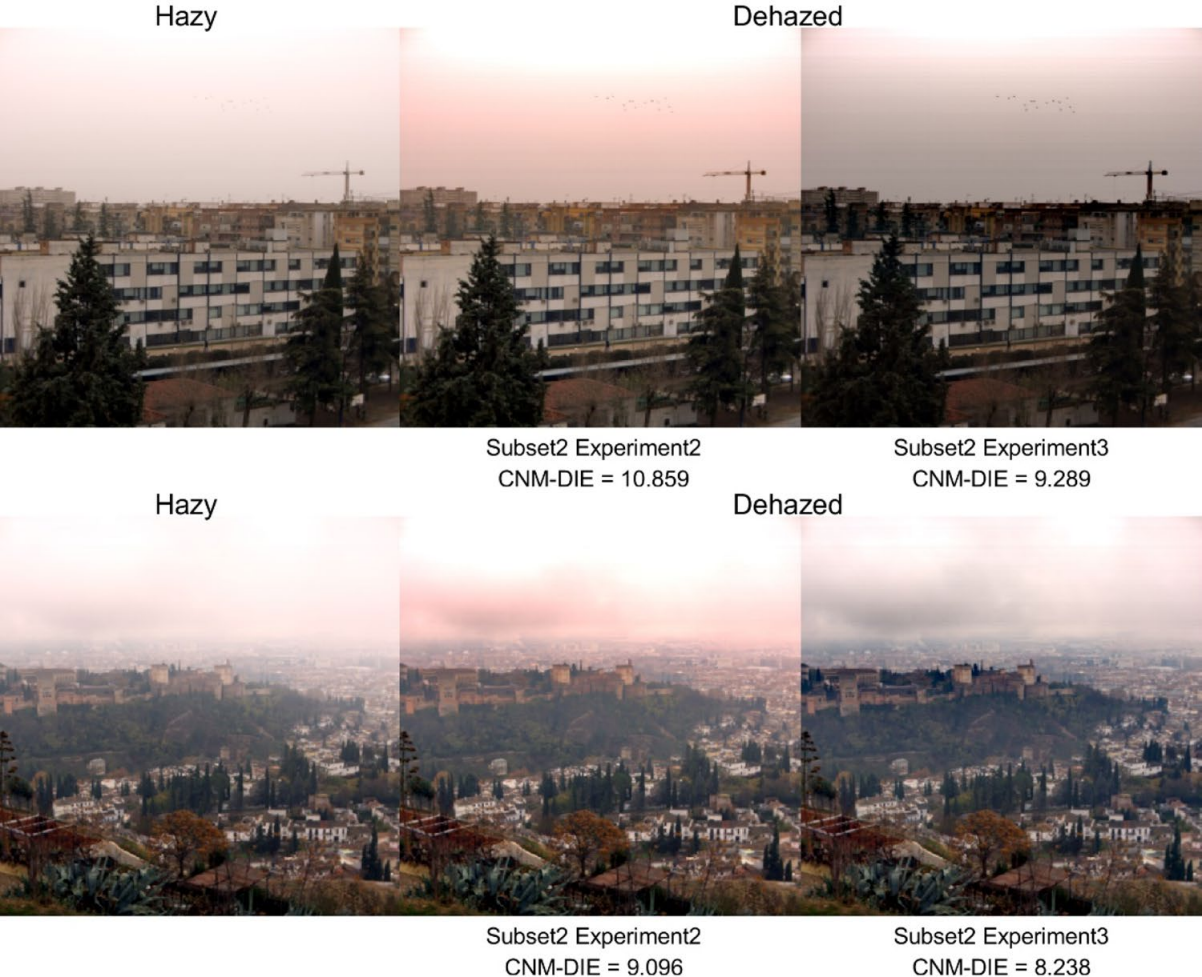
Comparison between results of experiments 2 and 3: example images not shown in previous figures.

In Fig. 9, we present some instances of the comparison between experiments 2 and 3 for images of subset 2. In the upper part of the figure, the contrast is noticeably improved and the color is more similar to the original hazy imagen for experiment 3 (i.e. the flock of birds in the sky, that are visible after dehazing, or the arm of the crane). For the scene in the lower part of Fig. 9, we notice improved visibility for the Alhambra and the houses beyond, while the details of the clouds are also more clearly defined. Again, the color is more natural and similar to the hazy image for experiment *3.*

## Conclusion

In this study, we have made two significant contributions to the field of outdoor hyperspectral image analysis. First, we introduce the GRANHHADA database, which, to the best of our knowledge, is the first outdoor hyperspectral image database featuring a combination of simulated and real hazy images. Our second contribution involves an exploration of dehazing methods using spectral information in both simulated and real hazy images. We evaluated three distinct approaches: optimal triplet band selection, simple dehazing using an sRGB rendition of the images, and mono-band spectral dehazing followed by sRGB rendering. Our findings reveal that the optimal triplet selection method consistently outperforms the other approaches across various scenarios.

Notably, the need for transformation between the optimal triplet and conventional RGB representation, which may be relevant in specific applications, does not diminish the significance of this method. In many fields, as outlined in Sect. 1, the color information may not be paramount.

For outdoor simulated hazy scenes (subset 1), our analysis demonstrates that optimal triplets tend to reside in the longer wavelengths region, with their selection influenced by the chosen metric. This observation highlights the necessity for efficient band reduction techniques, tailored for hyperspectral images, particularly in situations where dehazing is essential.

In the face of challenging worst-case scenarios, characterized by high haze densities and the presence of suspended dust particles unique to the GRANHHADA database, it's clear that the dehazing task remains complex, even for Deep-Learning based methods like MS-CNN^19^.

When dealing with real hazy images (subset 2), the benefits of optimal triplet selection are consistently more pronounced when compared to sRGB and mono-band plus sRGB approaches. Nevertheless, if the full spectral image is available, performing band-by-band dehazing prior to sRGB rendering can significantly enhance results, thereby underscoring the potential of hyperspectral images for dehazing applications.

Our work further underscores the value of optimal triplet searches as a preliminary step for reducing the number of bands in capture devices, as previously highlighted in our earlier research^26^. Moreover, our results advocate for the use of band-by-band dehazing algorithms, even when MS-CNN has not been specifically trained with monochrome images. This paves the way for exploring deep learning-based methods, particularly those trained with spectral images featuring high densities of real haze of different types^59,60^.

While we have made some progress in advancing the field of hyperspectral image dehazing, we also acknowledge intrinsic limitations in the evaluation process, particularly concerning image quality metrics. To this end, the development of non-reference metrics tailored specifically for dehazing could be a valuable asset. Such metrics should ideally encompass the effects induced by haze, including local contrast reduction, chroma loss, and overall scene brightness increase. Their application would facilitate a more comprehensive evaluation of the efficacy of dehazing methods in mitigating these adverse effects.

## Data Availability

The dataset generated and analysed the current study are available in our research group page in our research group page (Color Imaging Lab), [https://colorimaginglab.ugr.es/pages/Data#__doku_granada_hyperspectral_hazy_database_granhhada].

## References

[1] McCartney EJ (1976). Optics of the atmosphere: scattering by molecules and particles. New York.

[2] Narasimhan, S. G., Wang, C., & Nayar, S. K. All the images of an outdoor scene. In *Computer Vision—ECCV 2002: 7th European Conference on Computer Vision Copenhagen, Denmark, May 28–31, 2002 Proceedings, Part III 7* (pp. 148–162). Springer Berlin Heidelberg (2002). 10.1007/3-540-47977-5_10

[3] Liou, K. N. *An introduction to atmospheric radiation* (Vol. 84). Elsevier (2002). 10.1256/003590003102695746

[4] Pincus R (2004). A first course on atmospheric radiation..

[5] Gomes AE, Linhares JM, Nascimento SM (2020). Near perfect visual compensation for atmospheric color distortions. Color. Res. Appl..

[6] Wierzbicki D, Kedzierski M, Sekrecka A (2019). A method for dehazing images obtained from low altitudes during high-pressure fronts. Remote Sens..

[7] Mehra A, Mandal M, Narang P, Chamola V (2020). ReViewNet: A fast and resource optimized network for enabling safe autonomous driving in hazy weather conditions. IEEE Trans. Intell. Transp. Syst..

[8] Fan C, Peng Y, Peng S, Zhang H, Wu Y, Kwong S (2021). Detection of train driver fatigue and distraction based on forehead EEG: A time-series ensemble learning method. IEEE Trans. Intell. Transp. Syst..

[9] Xie Z, Li Y, Niu J, Shi L, Wang Z, Lu G (2020). Hyperspectral face recognition based on sparse spectral attention deep neural networks. Opt. Express.

[10] Cimtay Y (2021). Smart and real-time image dehazing on mobile devices. J. Real-Time Image Process..

[11] Jiang B, Chen G, Wang J, Ma H, Wang L, Wang Y, Chen X (2021). Deep dehazing network for remote sensing image with non-uniform haze. Remote Sens..

[12] Wang C, Hu J, Luo X, Kwan MP, Chen W, Wang H (2022). Color-dense illumination adjustment network for removing haze and smoke from fire scenario images. Sensors.

[13] Wang D, Qi J, Huang B, Noble E, Stoyanov D, Gao J, Elson DS (2022). Polarization-based smoke removal method for surgical images. Biomed. Opt. Express.

[14] Khan H, Xiao B, Li W, Muhammad N (2022). Recent advancement in haze removal approaches. Multimedia Syst..

[15] Nair D, Sankaran P (2022). Benchmarking single image dehazing methods. SN Comput. Sci..

[16] Hartanto CA, Rahadianti L (2021). Single image dehazing using deep learning. JOIV Int. J. Inf. Vis..

[17] Juneja A, Kumar V, Singla SK (2022). A systematic review on foggy datasets: applications and challenges. Arch. Comput. Methods Eng..

[18] Choi LK, You J, Bovik AC (2015). Referenceless prediction of perceptual fog density and perceptual image defogging. IEEE Trans. Image Process..

[19] Liu W, Zhou F, Lu T, Duan J, Qiu G (2020). Image defogging quality assessment: Real-world database and method. IEEE Trans. Image Process..

[20] Ma, K., Liu, W., & Wang, Z. Perceptual evaluation of single image dehazing algorithms. In *2015 IEEE International Conference on Image Processing (ICIP)* (pp. 3600–3604) (2015). IEEE. 10.1109/ICIP.2015.7351475

[21] Zhao, S., Zhang, L., Huang, S., Shen, Y., Zhao, S., & Yang, Y. Evaluation of defogging: A real-world benchmark dataset, a new criterion and baselines. In *2019 IEEE international conference on multimedia and expo (ICME)* (pp. 1840–1845) (2019). IEEE. 10.1109/ICME.2019.00316

[22] Zhao S, Zhang L, Huang S, Shen Y, Zhao S (2020). Dehazing evaluation: Real-world benchmark datasets, criteria, and baselines. IEEE Trans. Image Process..

[23] Jiao W, Jia X, Liu Y, Jiang Q, Sun Z (2021). Single image mixed dehazing method based on numerical iterative model and DehazeNet. PLoS ONE.

[24] Ngo D, Lee S, Lee GD, Kang B (2021). Automating a dehazing system by self-calibrating on haze conditions. Sensors.

[25] Lüthen J, Wörmann J, Kleinsteuber M, Steurer J (2017). A rgb/nir data set for evaluating dehazing algorithms. Electron. Imaging.

[26] El Khoury, J., Thomas, J. B., & Mansouri, A. A spectral hazy image database. In *Image and Signal Processing: 9th International Conference, ICISP 2020, Marrakesh, Morocco, June 4–6, 2020, Proceedings* (pp. 44–53). Cham: Springer International Publishing (2020). 10.1007/978-3-030-51935-3_5

[27] Fernández-Carvelo S, Martínez-Domingo MÁ, Valero EM, Romero J, Nieves JL, Hernández-Andrés J (2021). Band selection for dehazing algorithms applied to hyperspectral images in the visible range. Sensors.

[28] Sun, Z., Zhang, Y., Bao, F., Wang, P., Yao, X., & Zhang, C. Sadnet: Semi-supervised single image dehazing method based on an attention mechanism. *ACM Trans. Multimedia Comput. Commun. Appl. (TOMM)*, **18**(2), 1–23 (2022). 10.1145/3478457

[29] Zhang S, Zhang J, He F, Hou N (2023). DRDDN: Dense residual and dilated dehazing network. Vis. Comput..

[30] Miko Viitakoski. Specim IQ. https://www.specim.com/iq/

[31] Resonon Pika IR+ Hyperspectral Imaging Camera, https://resonon.com/Pika-IR-Plus

[32] Oakley JP, Satherley BL (1998). Improving image quality in poor visibility conditions using a physical model for contrast degradation. IEEE Trans. Image Process..

[33] Husain, N. A., Rahim, M. S. M., Kari, S., & Chaudhry, H. VRHAZE: The simulation of synthetic haze based on visibility range for dehazing method in single image. In *2020 6th International Conference on Interactive Digital Media (ICIDM)* (pp. 1–7) (2020). IEEE. 10.1109/ICIDM51048.2020.9339638

[34] Nayar, S. K., & Narasimhan, S. G. Vision in bad weather. In *Proceedings of the seventh IEEE international conference on computer vision* (Vol. 2, pp. 820–827) (1999). IEEE. 10.1109/ICCV.1999.790306

[35] Tarel JP, Hautiere N, Caraffa L, Cord A, Halmaoui H, Gruyer D (2012). Vision enhancement in homogeneous and heterogeneous fog. IEEE Intell. Transp. Syst. Mag..

[36] Zhang, Y., Ding, L., & Sharma, G. Hazerd: An outdoor scene dataset and benchmark for single image dehazing. In *2017 IEEE international conference on image processing (ICIP)* (pp. 3205–3209) (2017). IEEE. 10.1109/ICIP.2017.8296874

[37] Li Y, You S, Brown MS, Tan RT (2017). Haze visibility enhancement: A survey and quantitative benchmarking. Comput. Vis. Image Underst..

[38] Hsieh CH, Chang YH (2021). Improving DCP haze removal scheme by parameter setting and adaptive gamma correction. Adv. Syst. Sci. Appl..

[39] Wang, C., Huang, Y., Zou, Y., & Xu, Y. FWB-Net: Front white balance network for color shift correction in single image dehazing via atmospheric light estimation. In *ICASSP 2021–2021 IEEE International Conference on Acoustics, Speech and Signal Processing (ICASSP)* (pp. 2040–2044) (2021). IEEE. 10.1109/ICASSP39728.2021.9414200

[40] Panchenko MV, Kozlov VS, Polkin VV, Terpugova SA, Polkin VV, Uzhegov VN, Zenkova PN (2019). Aerosol characteristics in the near-ground layer of the atmosphere of the city of Tomsk in different types of aerosol weather. Atmosphere.

[41] Preiss J, Fernandes F, Urban P (2014). Color-image quality assessment: From prediction to optimization. IEEE Trans. Image Process..

[42] Wang, Z., Simoncelli, E. P., & Bovik, A. C. Multiscale structural similarity for image quality assessment. In *The Thrity-Seventh Asilomar Conference on Signals, Systems & Computers, 2003* (Vol. 2, pp. 1398–1402) (2003). 10.1109/ACSSC.2003.1292216

[43] Sharma G, Wu W, Dalal EN (2005). The CIEDE2000 color-difference formula: Implementation notes, supplementary test data, and mathematical observations. Color Res. Appl..

[44] Seshadrinathan, K., Pappas, T.N., Safranek, R. J., Chen, J., Wang, Z., Sheikh, H. R. & Bovik, A. C. “Image quality assessment,” in The Essential Guide to Image Processing, (Elsevier, 2009), pp. 553–595.

[45] Zhang L, Zhang L, Mou X, Zhang D (2011). FSIM: A feature similarity index for image quality assessment. IEEE Trans. Image Process..

[46] Zhang L, Shen Y, Li H (2014). VSI: A visual saliency-induced index for perceptual image quality assessment. IEEE Trans. Image Process..

[47] Sheikh HR, Bovik AC (2006). Image information and visual quality. IEEE Trans. Image Process..

[48] Hautiere N, Tarel JP, Aubert D, Dumont E (2008). Blind contrast enhancement assessment by gradient ratioing at visible edges. Image Anal. Stereol..

[49] Fang, S., Yang, J., Zhan, J., Yuan, H., & Rao, R. Image quality assessment on image haze removal. In *2011 Chinese Control and Decision Conference (CCDC)* (pp. 610–614) (2011). IEEE. 10.1109/CCDC.2011.5968254

[50] Guo F, Tang J, Cai ZX (2014). Objective measurement for image defogging algorithms. J. Cent. South Univ..

[51] Yang D, Shen Y, Shen Y, Li H (2016). Reduced-reference image quality assessment using moment method. Int. J. Electron..

[52] Moulden B, Kingdom F, Gatley LF (1990). The standard deviation of luminance as a metric for contrast in random-dot images. Perception.

[53] Mittal A, Moorthy AK, Bovik AC (2012). No-reference image quality assessment in the spatial domain. IEEE Trans. Image Process..

[54] Mittal A, Soundararajan R, Bovik AC (2012). Making a “completely blind” image quality analyzer. IEEE Signal Process. Lett..

[55] Venkatanath, N., Praneeth, D., Bh, M. C., Channappayya, S. S. & Medasani, S. S. Blind image quality evaluation using perception based features. In *2015 twenty first national conference on communications (NCC)* (pp. 1–6). IEEE (2015). 10.1109/NCC.2015.7084843

[56] Tang, H., Joshi, N. & Kapoor, A. Learning a blind measure of perceptual image quality. In *CVPR 2011* (pp. 305–312) (2011). IEEE. 10.1109/CVPR.2011.5995446

[57] Moorthy AK, Bovik AC (2011). Blind image quality assessment: From natural scene statistics to perceptual quality. IEEE Trans. Image Process..

[58] Silberman N, Hoiem D, Kohli P, Fergus R (2012). Indoor segmentation and support inference from rgbd images. ECCV.

[59] Bianco, S., Celona, L. & Piccoli, F. Single image dehazing by predicting atmospheric scattering parameters. In *London Imaging Meeting* (Vol. 2020, No. 1, pp. 74–77). Society for Imaging Science and Technology (2020). 10.2352/issn.2694-118X.2020.LIM-11

[60] Wang, J., Ding, C., Wu, M., Liu, Y., & Chen, G. A brief review of image dehazing algorithms based on deep learning. In *The International Conference on Image, Vision and Intelligent Systems (ICIVIS 2021)* (pp. 377–391) (2022). Singapore: Springer Nature Singapore.

